# Identifying high occurrence areas of hospitalization and mortality from respiratory diseases in the Brazilian Legal Amazon: a space-time analysis

**DOI:** 10.1590/0102-311XEN148023

**Published:** 2024-11-25

**Authors:** Lucas de Oliveira do Couto, Ludmilla da Silva Viana Jacobson, Andre Reynaldo Santos Périssé, Sandra de Souza Hacon

**Affiliations:** 1 Escola Nacional de Saúde Pública Sergio Arouca, Fundação Oswaldo Cruz, Rio de Janeiro, Brasil.; 2 Universidade Federal Fluminense, Niterói, Brasil.

**Keywords:** Respiratory Diseases, Hospitalization, Amazonian Ecosystem, Doenças Respiratórias, Hospitalização, Ecosistema Amazônico, Enfermedades Respiratorias, Hospitalización, Ecosistema Amazónico

## Abstract

Respiratory diseases pose a significant threat to the health of the Brazilian population, ranking among the leading causes of hospitalizations and deaths in the country. The most impacted demographics are children, adolescents, and older adults, who respectively have the highest rates of hospitalizations and deaths. An exploratory ecological study was conducted to assess the spatio-temporal distribution of hospitalizations and deaths due to respiratory diseases among children, adolescents, and older adults residing in municipalities in the Brazilian Legal Amazon. Moreover, the study aimed to identify priority municipalities within the detected clusters by employing composite synthetic municipal indices. These indices were estimated based on various socio-environmental and health indicators. The scan analysis identified clusters across various time periods but they mostly aligned with the disease trends in the region. We were able to identify clusters both near metropolitan areas and in remote locations, capturing two distinct patterns of cluster distribution. Moreover, the application of composite synthetic indices enabled a comprehensive identification of priority municipalities, considering various factors relevant to the health conditions of the population in the studied areas.

## Resumo

As doenças respiratórias representam uma ameaça significativa à saúde da população brasileira, estando entre as principais causas de hospitalizações e mortes no país. As populações mais impactadas são crianças, adolescentes e idosos, que apresentam, respectivamente, as maiores taxas de hospitalizações e óbitos. Foi realizado um estudo ecológico exploratório para avaliar a distribuição espaço-temporal das hospitalizações e óbitos por doenças respiratórias entre crianças, adolescentes e idosos residentes em municípios da Amazônia Legal. Além disso, o estudo teve como objetivo identificar os municípios prioritários dentro dos *clusters* detectados, empregando índices municipais sintéticos compostos. Esses índices foram calculados a partir de diversos indicadores socioambientais e de saúde. A análise de varredura identificou *clusters* em uma variedade de períodos de tempo, mas, em geral, eles se alinham com as tendências de doenças na região. Foi possível identificar *clusters* perto de áreas metropolitanas e em locais remotos, capturando dois padrões distintos de distribuição de *clusters*. Além disso, a aplicação de índices sintéticos compostos possibilitou a identificação abrangente dos municípios prioritários, considerando uma variedade de fatores relevantes para as condições de saúde da população nas áreas estudadas.

## Resumen

Las enfermedades respiratorias representan una importante amenaza para la salud de la población brasileña y figuran entre las principales causas de hospitalizaciones y muertes en el país. Las poblaciones más afectadas son los niños, los adolescentes y los ancianos, que presentan, respectivamente, las tasas más altas de hospitalizaciones y muertes. Se realizó un estudio ecológico exploratorio para evaluar la distribución espacio-temporal de las hospitalizaciones y muertes por enfermedades respiratorias entre niños, adolescentes y ancianos residentes en municipios de la Amazonia Legal. Además, el estudio tuvo como objetivo identificar los municipios prioritarios dentro de los *clusters* detectados, empleando índices municipales sintéticos compuestos. Estos índices se calcularon con base en diversos indicadores socioambientales y de salud. Análisis de escaneo identificado *clusters* en una variedad de períodos de tiempo, pero, en general, se alinean con las tendencias de enfermedades en la región. Fue posible identificar *clusters* cerca de áreas metropolitanas y en ubicaciones remotas, capturando dos patrones distintos de distribución de *clusters*. Además, la aplicación de índices sintéticos compuestos permitió la identificación integral de municipios prioritarios, considerando una variedad de factores relevantes a las condiciones de salud de la población en las áreas estudiadas.

## Introduction

Respiratory diseases rank among the leading causes of hospitalizations and deaths across Brazil, serving as the second major cause of hospitalizations and the fourth leading cause of deaths in the country ^1^. From 2000 to 2019, the Brazilian Legal Amazon recorded over 1.7 million hospitalizations for individuals aged from 1 to 19 years, with pneumonia and asthma being the predominant causes ^2^. Throughout the same period, the region recorded the loss of more than 124,000 older adults aged 60 or older, primarily due to pneumonia and chronic diseases of the lower respiratory tract ^3^.

Data from the Brazilian Hospital Information System of the Brazilian Unified National Health System (SIH-SUS) reveal that the mortality rates among older adults hospitalized for respiratory diseases have been increasing across Brazil since 2009, including in the Brazilian Legal Amazon. Moreover, despite the number of hospitalizations of children and adolescents in Brazilian Legal Amazon states decreased during the same period, a comparative analysis with other Brazilian states indicated an increase in mortality among children and adolescents hospitalized for respoiratory diseases ^2^.

The incidence of hospitalizations and deaths due to respiratory diseasesd is associated with various risk factors, including exposure to viral and bacterial agents, allergens, atmospheric pollutants, and climatic conditions ^4^ ^,^ ^5^. Physiological and socio-economic factors also influence the resilience of exposed individuals, meaning that the severity of health outcomes often results from the interplay of diverse risk factors and health determinants ^4^ ^,^ ^6^.

A critical aspect in the Brazilian Leal Amazon is the occurrence of fires and wildfires, predominantly during the driest months from July to November. These fires serve as significant sources of fine particulate matter (PM_2.5_) in the region, an atmospheric pollutant widely recognized for its adverse health effects. Deforestation in the region plays a significant role in this scenario. From 2009 to 2018, the average annual deforestation rate was 6,490km^2^, peaking at 7,893km^2^ in 2016. From 2019 to 2022, there was a notable increase in deforestation, peaking at 13,000km^2^ in 2021, with an annual average rate of 11,400km^2^. The latest data for 2023 show a decline to 9,000km^2^, marking the lowest rate since 2018 ^7^ ^,^ ^8^.

The region shows an uneven distribution of risk factors, such as those aforementioned, and other health determinants associated with the occurrence and exacerbation of respiratory diseases in children, adolescents, and older adults ^9^. Certain areas may exhibit conditions more or less conducive to the development and exacerbation of these diseases. Therefore, understanding the spatial distribution of these diseases in the Brazilian Legal Amazon is critical for identifying risk factors, defining priority areas, and strategizing control and prevention actions ^10^.

In this study, we employed a spatio-temporal scan method (SaTScanTM; http:\\www.satscan.org) across the 772 municipalities of the Brazilian Legal Amazon to detect and assess the significance of spatio-temporal clusters with high occurrences of hospitalizations for respiratory diseases in individuals aged from 1 to 19 years, as well as high mortality risk clusters in older adults aged over 60 years. Additionally, we estimated municipal composite synthetic indices to evaluate the profile of each municipality within the detected clusters based on various socio-environmental and health indicators. We anticipate that this combined approach will aid in identifying priority areas and facilitate the formulation of effective control and prevention strategies for these diseases.

## Method

### Study design and area: Brazilian Legal Amazon

An exploratory ecological study was conducted using the Kulldorff’s space-time scan statistic (SaTScanTM; http:\\www.satscan.org) to assess the monthly spatio-temporal distribution of hospitalizations and deaths due to respiratory diseases across 772 municipalities in the Brazilian Legal Amazon from 2009 to 2019. Additionally, municipal composite synthetic indices were developed based on socio-environmental and health indicators, aiming to better characterize and prioritize the municipalities detected in the cluster analyses of each state and outcome.

The Brazilian Legal Amazon spans an area of 5,016,478.27km^2^ of the national territory and encompasses nine states: Acre, Amazonas, Amapá, Mato Grosso, Pará, Rondônia, Roraima, Tocantins, and part of Maranhão. The region features a mix of some densely populated urban areas along with generally low-density urban and rural areas. Many of the municipalities within the region show populations of fewer than 50,000 residents. The Municipal Human Development Index (M-HDI) in the states of the Brazilian Legal Amazon ranges from 0.68 to 0.74 ^7^.

The region experiences two distinct seasons: the rainy season, from December to May, and the dry season, from July to October. Historically, most fires occur during the dry season, with the period from July to November being the most critical. The region’s climate is hot and humid, with an average temperature of 25.7ºC and a 88.1% humidity during the rainy season, and 26.4ºC and 77.2% during the dry season ^11^.

### Study population and outcome analyzed

The study focused on two age groups: children and adolescents (aged from 1 to 19 years) and older adults (aged 60 and older). Hospitalizations due to respiratory diseases in children and adolescents were evaluated, along with deaths from respiratory diseases among older adults. These age groups were chosen based on their inherent susceptibilities to the studied outcomes, such as the developing immune and respiratory systems in children and adolescents, and the higher prevalence of chronic diseases and diminished immune response in older adults ^4^ ^,^ ^12^.

### Data source and collection

#### SUS informatics system

Monthly data on hospitalizations and deaths due to respiratory diseases were collected from the Brazilian Health Informatics Department (DATASUS). All cases were gathered based on the individuals’ municipality of residence, month of occurrence, and specific cause. The following data were included: hospitalizations for individuals aged from 1 to 19 years, and deaths for people aged 60 or older, following Chapter X of the International Classification of Diseases, 10th revision (ICD-10). Municipal population estimates for each year from 2009 to 2019, stratified by age group, were obtained from the annual projections provided by DATASUS. The collection and organization of data were conducted using the R environment, version 4.1 (http://www.r-project.org).

#### Social and environmental indicators for municipality prioritization

Social, environmental, and health indicators were used to construct synthetic indexes, which subsequently assisted in prioritizing municipalities identified in the scan analysis. Some of these indicators were directly sourced from the Amazon SPI 2021 (Amazon Social Progress Index) ^13^ dataset, with municipality-specific data available at: https://ipsamazonia.org.br.

Estimations of monthly PM_2.5_ concentrations in municipalities within the Brazilian Legal Amazon were obtained by the Atmospheric Composition Analysis Group at the University of Washington, using globally available NetCDF format data with a 1km spatial resolution ^14^ ^,^ ^15^. The database was structured using the QGIS software (https://qgis.org/en/site/) and the R environment (http://www.r-project.org). A comprehensive description of the index is provided in a following specific section.

#### Space-time cluster analysis

The Kulldorff’s space-time scan was employed to identify municipalities with a high monthly incidence of morbidity and mortality from respiratory diseases in the states of the Brazilian Legal Amazon. This method was chosen as it (i) detects space-time clusters, minimizing pre-selection bias by searching for clusters without specifying their size or location; (ii) tests the statistical significance of clusters via Monte Carlo simulations, ordering them according to the likelihood ratio test; (iii) examines disease dynamics in continuous time and can adjust for population density, dealing with non-homogeneous populations; (iv) estimates the relative risk (RR) for each cluster, considering the underlying population (http:\\www.satscan.org).

The scan traverses the entire study area (each state of the Brazilian Legal Amazon) using a cylindrical window (*z*) with a variable radius (space) and height (time). The window expands from the centroid of each municipality, detecting clusters by contrasting the expected and observed number of events within these z-windows (http:\\www.satscan.org).

The null hypothesis states that there are no clusters in studied area, implying that the risk of hospitalizations and deaths from respiratory diseases is identical both inside and outside the scanning window (*z*).

In this study, scans were performed individually in each state of the Brazilian Legal Amazon. The model used was based on the Poisson distribution, which considers both the number of cases and the estimated population size in each municipality for the analysis. In this model, the expected number of cases *E[c]* within a window z is estimated by:

$E\left[c\right]=(\frac{C}{P})\times p$ (1)

In this context, *C* represents the total number of cases in the state, *P* stands for the total population of the state, and *p* the population within the scanning window *z*, which could refer to one municipality or multiple municipalities.

The estimation of the expected number of cases within a window z uses the total values of cases and resident population of the respective state to which the municipalities belong as references.

Significant clusters detected were ranked into primary, secondary, or higher order clusters, based on the log likelihood ratio. While primary clusters are the most statistically significant, secondary and higher order clusters also represent statistically significant findings. For a Poisson distribution data, the likelihood function of each z-cylinder is given by:

$(\frac{c}{E\left[c\right]}{)}^{c}(\frac{C-c}{C-E\left[c\right]}{)}^{C-c}$ (2)

In this context, *c* refers to the number of observed cases within the scanning window z, whereas *E[c]* is the expected number of cases within the same window. The term *C* represents the total number of cases within the analyzed state. Statistical significance is determined using Monte Carlo simulations.

The RR of hospitalizations and deaths, which represents the estimated risk within the cluster divided by the estimated risk outside the cluster, is estimated as follows:

$\text{RR=}\frac{c/E\left[c\right]}{\left(C-c)/(C-E\left[c\right]\right)}$ (3)

A circular scanning window was employed, with the maximum limit of the cluster set at 50% of the exposed population, following literature recommendations. Similarly, the maximum temporal window was set to be equal to 50% of the study period. Statistically significant clusters were defined as those with a p-value less than 0.05, obtained via 999 iterations of Monte Carlo simulations. The analyses were conducted using the SatScan software, version 10.1.

#### Prioritization of municipalities in detected clusters

In this study, the scan analysis technique was used to detect clusters of respiratory diseases in all municipalities of the Brazilian Legal Amazon. To prioritize these municipalities, a classification method was employed, incorporating socio-environmental and health indicators. These indicators were combined to create municipal composite synthetic indices, unique to the clusters detected in each state and for each analyzed outcome ^13^ ^,^ ^16^.

The municipal composite synthetic indices encompass various relevant socio-environmental indicators that influence the population’s health status, thereby facilitating a comprehensive assessment of each municipality’s performance and prioritization within the identified clusters.

In this analysis, a set of 23 municipal indicators were used, detailed in Box 1. In total, 17 of these were directly sourced from the SPI 2021 ^16^. Additionally, five indicators, namely hospitalizations and deaths rates due to respiratory diseases, relative risk, cluster order, and population density, were derived from the cluster analysis dataset and results of each outcome. The hospitalization rates were integrated into the indices of municipalities identified via hospitalization scans, whereas the death rates were incorporated into the indices of municipalities identified via death scans. Moreover, data on the estimated average concentration of PM_2.5_ was obtained from the Atmospheric Composition Analysis Group at the University of Washington (United States) ^14^ ^)^ and van Donkelaar et al. ^15^. A comprehensive variable dictionary from SPI 2021 is available on the project website ^13^.

**Box 1:** Municipal composite synthetic index.

INDICATOR	PILLAR	DIMENSION	
Dropout rate in elementary education * Grade-age distortion in elementary education * Grade-age distortion in high school *	Education	Education, Housing, and Sanitation	Index
Housing with adequate lighting * Housing with adequate walls * Housing with adequate flooring * Population density **^,###^	Housing		
Adequate water supply * Adequate sewage system * Proper garbage collection *	Sanitation		
Fine particulate matter (PM_2.5_) ***^,§^ Protected areas * Accumulated deforestation * Recent deforestation *	Environmental quality	Health and Environment ^##^	
Hospitalization/Death rate per 100,000 inhabitants **^,###^ Relative risk **^,###^ Cluster order **^,###^	Health − cluster ^#^		
Infant mortality under 5 years of age * Maternal mortality * Undernutrition * Cancer mortality * Respiratory disease mortality *	Health − overall

Source: prepared by the authors, with data from *SPI Amazon 2021* (Amazon Social Progress Index, https://painel.ipsamazonia.org.br/uploads/IPS_Amazonia_2023_dc7f4721ef.xlsx); Atmospheric Composition Analysis Group ^14^ and van Donkelaar et al. ^15^; Brazilian Health Informatics Department (https://datasus.saude.gov.br/informacoes-de-saude-tabnet/).

Data derived from: * SPI 2021 indicators, ** Cluster Analysis Dataset and Results, *** Atmospheric Composition Analysis Group.

^#^ Indicators with weight 2;

^##^ Dimension with weight 1.5;

^###^ Population density: average population density from 2009 to 2019; Hospitalization and death rates per 100,000 inhabitants: averages of annual rates estimated for each year from 2009 to 2019;

^§^ Fine particulate matter: average of monthly concentrations of PM_2.5_ from 2009 to 2019; indicators derived from SPI 2021 are described in: SPI Amazon (https://ipsamazonia.org.br/relatorios).

A linear transformation was employed using the min-max normalization method to rescale the indicators, mapping their values on a scale of 0 to 100.

$X^{'}=\frac{x-x_{\text{min}}}{x_{\text{max}}-x_{\text{min}}}\left(u-l\right)\text{+l}$ (4)

In which *X’* is the normalized value, *x* is the original value of the indicator, *x*_*min*_ and *x*_*max*_ are the minimum and maximum values of the indicator, respectively, and *u* and *l* are the new dimensions with *l* = 0 and *u* = 100. The data is normalized on a scale of 0 to 100. The first term of the equation normalizes the values on the standard scale (0.1). The terms *u* and *l* are responsible for the customized scale (0-100). The term *+l* is more relevant in customized scales that do not start from zero. When *l* is different from zero, the term shifts the normalized variable within the new range, ensuring that the minimum value is equal to *l*.

After normalization, the indicators were grouped into six pillars, then into two dimensions, and finally into an index (Box 1). Data aggregation was performed via weighted arithmetic average among the indicators within each of their respective pillars, dimensions, and subsequently within the index, using the equation:

$\text{y=}\frac{1}{\sum_{\text{i=}1}^{d}w_{i}}\sum_{\text{i=}1}^{d}x_{i}w_{i}$ (5)

In which *w*_*i*_ represents the weights assigned to each component *x*_*i*_ . In our analysis, the indicators belonging to the “Health-cluster” pillar represent the most relevant variables in this index, as they are directly related to cluster analysis and were assigned a weight of 2. The Health and environment dimension, which combines information on important environmental indicators related to respiratory diseases and health data, including cluster data, received a weight of 1.5. The remaining indicators, pillars, and dimensions were assigned a weight of 1.

The scores ranged from 0 to 100, with higher values representing more favorable scenarios. Therefore, priority municipalities are those with lower indices.

## Results

**Figure 1:**
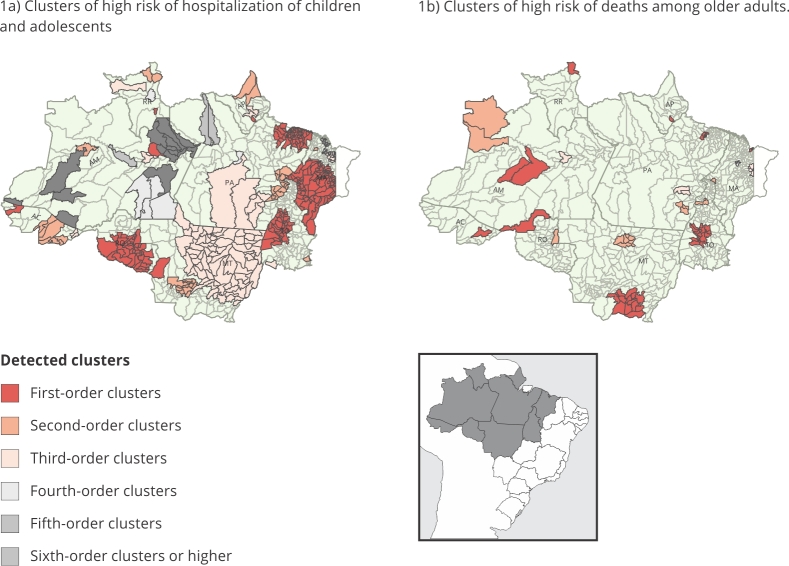
Space-time clusters detected in the Brazilian Legal Amazon from January 2009 to December 2019.

AC: Acre State; AM: Amazônia State; AP: Amapá State; MA: Maranhão State; MT: Mato Grosso State; RO: Rondônia State; RR: Roraima State; TO: Tocantins State. Source: prepared by the authors, with data from the Brazilian Health Informatics Department ^2^ ^,^ ^3^, Brazilian National Institute for Space Research ^8^.

The analysis revealed clusters with a high occurrence of hospitalizations and deaths from respiratory diseases in all states of the Brazilian Legal Amazon from 2009 to 2019. All of these clusters were statistically significant. We detected a total of 39 clusters with a high occurrence of hospitalization for respiratory diseases in children and adolescents, which are visually represented in Figure 1a. Additionally, we identified 19 clusters showing a high occurrence of death from respiratory diseases in older adults, as depicted in Figure 1b.

### Areas with high occurrences of hospitalization among children and adolescents

Table 1 depicts the areas with high occurrences of hospitalization among children and adolescents, providing insights into the characteristics of each identified cluster. Additionally, Figure 1a visually displays the locations of these clusters. Notably, Amazonas stands out with the highest number of identified clusters, totaling 12. Among them, Manaus emerges as a first-order cluster, exhibiting a relative risk of hospitalization 1.98 times higher than other municipalities in the state from April 2009 to September 2014. In Acre, three clusters were detected, with Cruzeiro do Sul being the first-order cluster, experiencing the highest estimated hospitalization risk of 3.82 among all clusters identified in the state. In Amapá, three clusters were identified, with the most recent one showing a relative risk of 2.13 from April to December 2019 (Table 1).

**Table 1:** Clusters of hospitalizations for children and adolescents due to respiratory diseases. Brazilian Legal Amazon, 2009-2019.

State/Cluster order	Cluster period	Municipalities (n)	Observed hospitalizations (n)	Expected hospitalizations (n)	RR
Acre					
1	Apr/2009 - Sep/2014	1	2,984	904	3.82
2	Apr/2009 - Sep/2014	7	2,617	1,234	2.34
3	Jan/2011 - Jun/2016	2	804	397	2.08
Amazonas					
1	Apr/2009 - Sep/2014	1	29,714	18,342	1.98
2	Jan/2009 - Dec/2011	1	491	168	2.94
3	Apr/2017 - Mar/2019	2	939	563	1.68
4	Jan/2009 - Jun/2010	3	568	290	1.97
5	Apr/2010 - Dec/2010	1	125	42	3.01
6	Jan/2009 - Mar/2013	1	279	154	1.81
7	Jan/2009 - Jun/2011	1	814	595	1.37
8	Apr/2012 - Jun/2016	1	430	285	1.51
9	Jul/2017 - Jun/2019	1	251	168	1.50
10	Apr/2010 - Jun/2010	9	196	124	1.58
11	Jul/2009 - Dec/2009	1	34	10	3.26
12	Jul/2017 - Mar/2018	1	56	27	2.10
Amapá					
1	Apr/2011 - Jun/2016	1	2,190	1,388	1.65
2	Apr/2019 - Dec/2019	2	144	68	2.13
3	Apr/2011 - Jun/2016	2	30	16	1,86
Maranhão					
1	Jan/2010 - Jun/2015	99	63,709	45,861	1.57
2	Jul/2010 - Dec/2015	1	2,475	462	5.41
3	Apr/2009 - Sep/2014	14	9,937	6,161	1.64
4	Apr/2009 - Jun/2014	4	2,673	1,624	1.65
5	Apr/2009 - Sep/2014	1	882	351	2.52
6	Jan/2013 - Jun/2018	4	1,355	800	1.70
Mato Grosso					
1	Oct/2013 - Mar/2019	1	4,493	276	17.16
2	Jan/2009 - Jun/2014	13	6,879	2,738	2.65
3	Jan/2009 - Jun/2010	75	6,562	4,477	1.50
Pará					
1	Jan/2009 - Jun/2013	56	93,783	61,835	1.73
2	Jan/2009 - Jun/2014	15	16,556	6,902	2.48
3	Jan/2009 - Jun/2014	7	13,230	6,117	2.21
4	Jan/2009 - Mar/2014	1	1,274	304	4.20
5	Jan/2009 - Jun/2013	2	2,750	2,009	1.37
Rondônia					
1	Apr/2009 - Sep/2014	36	21,150	14,242	1.75
Roraima					
1	Apr/2010 - Dec/2013	1	319	60	5.43
2	Apr/2009 - Sep/2010	2	283	95	3.01
3	Jan/2012 - Jun/2017	1	550	265	2.12
Tocantins					
1	Jan/2009 - Jun/2014	38	8,822	4,839	2.04
2	Apr/2009 - Jun/2014	1	995	218	4.66
3	Apr/2009 - Sep/2014	1	632	110	5.82

RR: relative risk.

Source: prepared by the authors, with data from the Brazilian Health Informatics Department ^2^.

Note: all detected clusters are statistically significant, p-value < 0.001.

In Mato Grosso, three clusters were identified, with the first-order cluster standing out for both its significantly high relative risk (RR = 17.16) and its remarkable duration, spanning from October 2013 to March 2019. Moving to Pará, a first-order cluster formed by 56 municipalities from January 2009 to June 2013 exhibited an RR = 1.73. In Tocantins, three clusters were also detected, with the highest-risk cluster (RR = 5.82) being a third-order cluster identified from April 2009 to September 2014 (Table 1).

In Maranhão State, the largest cluster was observed, encompassing 99 municipalities from January 2010 to June 2015, with a RR = 1.57. Notably, the highest-risk cluster (RR = 5.41) was confined to a single municipality in the northern region, during the same period as the previous one (Table 1). In Roraima State, the main cluster solely involved the municipality of São Luiz, revealing a RR = 5.43 from April 2010 to December 2013. As for Rondônia State, a cluster comprising 36 municipalities was detected during the same period, exhibiting a RR = 1.75 (Table 1).

### Areas with a high occurrence of deaths among older adults

Clusters of deaths from respiratory siseases in older adults have been identified across several states, albeit in smaller numbers compared to hospitalization clusters. Table 2 provides details on the characteristics of each of these clusters and Figure 1b visually displays their locations.

**Table 2:** Mortality clusters for older adults due to respiratory diseases. Brazilian Legal Amazon, 2009-2019.

State/Cluster order	Cluster period	Municipalities (n)	Observed deaths	Expected deaths	RR
Acre					
1	Jan/2014 - Jun/2019	1	1,225	971	1.39
Amazonas					
1	Jan/2015 - Dec/2019	2	275	166	1.68
2	Oct/2016 - Dec/2019	2	98	42	2.33
3	Oct/2014 - Sep/2016	1	88	50	1.77
Amapá					
1	Apr/2015 - Jun/2019	1	195	135	1.5
Maranhão					
1	Apr/2014 - Sep/2019	4	3,830	2,082	2.09
2	Apr/2013 - Dec/2017	1	537	334	1.63
3	Apr/2015 - Dec/2019	5	598	401	1.51
4	Jan/2017 - Jun/2019	4	167	107	1.57
Mato Grosso					
1	Apr/2012 - Mar/2017	15	3,223	2,522	1.36
2	Jul/2014 - Dec/2019	6	287	203	1.43
Pará					
1	Apr/2014 - Sep/2019	7	8,301	5,447	1.74
2	Jan/2017 - Dec/2019	3	164	104	1.58
3	Oct/2017 - Sep/2019	2	136	83	1.63
Rondônia					
1	Jan/2012 - Jun/2017	1	1,037	719	1.53
2	Apr/2009 - Sep/2012	1	290	135	2.21
Roraima					
1	Jan/2014 - Dec/2016	1	13	4	3.31
Tocantins					
1	Apr/2012 - Mar/2016	20	617	438	1.47
2	Oct/2012 - Jun/2015	2	185	118	1.59

RR: relative risk.

Source: prepared by the authors, with data from the Brazilian Health Informatics Department ^3^.

Note: all detected clusters are statistically significant, p-value < 0.05.

Among these states, Maranhão, Amazonas, and Pará stood out with the highest number of clusters, recording four, three, and three clusters, respectively. The first-order clusters in these states were observed from 2014 to 2019. In contrast, Tocantins and Mato Grosso states exhibited larger clusters, encompassing 20 municipalities in Tocantins (RR = 1.47) and 15 municipalities in Mato Grosso State (RR = 1.36). The first-order clusters in these states were recorded from 2012 to 2017. As for Roraima, the first-order cluster with the highest estimated relative risk of respiratory diseases death (RR = 3.31) covered only one municipality and was detected from January 2014 to December 2016 (Table 2).

### Synthetic indexes and priority municipalities

**Figure 2:**
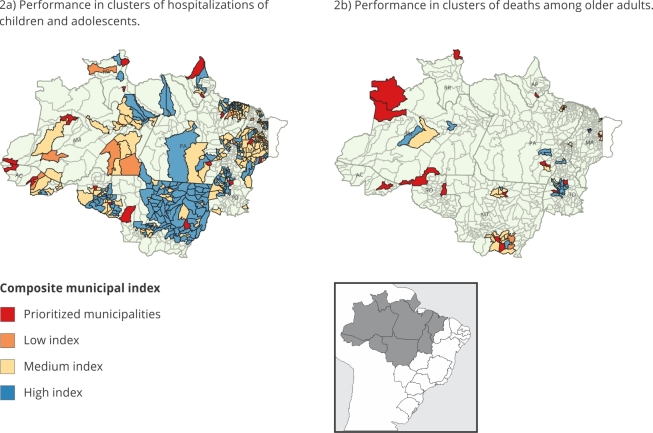
Municipal composite socioenvironmental index.

Figures 2a and 2b visually represent the estimated composite synthetic indices, highlighting in red the priority municipalities, which are those with the lowest indices. Tables 3 and 4 present an overview of the municipalities prioritized in the study, illustrating each composite index score, the statewide index range across municipalities, and their performance in specific dimensions, namely, Education, Housing and Sanitation, and Health and Environment. Additionally, the tables display the cluster order for each municipality, along with the respective RR and hospitalization rate.

AC: Acre State; AM: Amazônia State; AP: Amapá State; MA: Maranhão State; MT: Mato Grosso State; RO: Rondônia State; RR: Roraima State; TO: Tocantins State. Source: prepared by the authors, with data from the Brazilian Health Informatics Department_^(2)^_ ; SPI Amazon 2021 (Amazon Social Progress Index, https://painel.ipsamazonia.org.br/uploads/IPS_Amazonia_2023_dc7f4721ef.xlsx); Atmospheric Composition Analysis Group _^(14)^_ and Van Donkelaar et al. _^(15)^_ ; Brazilian National Institute for Space Research _^(8)^_ . Note: in 2b, the states of Acre, Roraima, and Amapá included only one municipality in the scan analysis and thus did not have their indices estimated.

**Table 3:** Priority municipalities selected from respiratory diseases hospitalization clusters. Brazilian Legal Amazon, 2009-2019.

State/Municipality	Municipal index	State index range	Education, Housing and Sanitation Dimension	Health and Environment Dimension	Cluster order	RR	Hospitalization rate
Acre							
Santa Rosa do Purus	40.3	40.3-76.6	11.4	59.6	2	1.9	681
Cruzeiro do Sul	42.2	-	62.8	28.4	1	3.8	951
Amazonas							
Fonte Boa	38.7	38.7-81.8	43.9	35.2	2	2.9	712
Guajará	48.9	-	59.9	41.7	6	1.8	546
Amapá							
Santana	47.7	47.7-63.9	81.2	25.4	1	1.6	886
Oiapoque	49.5	-	31.8	61.3	2	2.2	408
Maranhão							
São Félix de Balsas	40.9	40.9-73.3	36.4	43.9	1	3.3	2,224
Arame	45.9	-	48.3	44.3	1	2.5	1,926
Mato Grosso							
Comodoro	44.8	44.8-85.8	61.7	33.6	1	17.2	7,894
Campinápolis	61.2	-	31.1	81.2	3	1.4	960
Pará							
Bannach	38.8	38.8-68.5	53.8	28.7	3	5.9	3,732
Igarapé-Miri	46.1	-	44.2	47.4	1	2.8	2,557
Rondônia							
Santa Luzia D’Oeste	38.5	38.5-77.0	62.77	22.26	1	3.23	4,482
Monte Negro	44.8	-	51.67	40.01	1	3.02	1,843
Roraima							
São Luiz	48.2	48.2-65.1	87.29	22.14	1	5.43	1,865
Normandia	52.6	-	27.47	69.41	2	3.45	898
Tocantins							
Tocantínia	35.9	35.9-74.4	15.35	49.62	1	4.58	2,445
Oliveira de Fátima	51.6	-	55.50	49.07	1	1.12	1,236

RR: relative risk.

Source: prepared by the authors, with data from: Brazilian Health Informatics Department ^(^ ^2^; SPI Amazon 2021 (Amazon Social Progress Index, https://painel.ipsamazonia.org.br/uploads/IPS_Amazonia_2023_dc7f4721ef.xlsx); Atmospheric Composition Analysis Group ^14^ and van Donkelaar et al. ^15^; Brazilian National Institute for Space Research ^8^.

Note: hospitalization rate per 100,000 inhabitants; State index range indicates the range of municipal index values within the state.

**Table 4:** Priority Municipalities selected from respiratory diseases mortality clusters. Brazilian Legal Amazon, 2009-2019.

State/Municipality	Municipal index	Index range	Education, Housing, and Sanitation Dimension	Health and Environment Dimension	Cluster order	RR	Mortality rate
Amazonas							
Japurá	34.7	34.9-73.4	46.5	27.1	2	3.2	528
São Gabriel da Cachoeira	51.5	-	36.9	61.2	2	2.3	508
Maranhão							
Peritoró	42.4	42.4-97.3	22.0	56.0	3	1.3	257
Bela Vista do Maranhão	44.4	-	41.9	46.0	4	1.9	310
Mato Grosso							
Santo Antônio do Leverger	45.7	45.7-67.3	39.6	49.8	1	1.0	417
Nova Santa Helena	49.7	-	62.2	41.4	2	1.6	466
Pará							
Bannach	40.6	40.6-74.9	31.9	46.3	2	1.8	340
Pau D’Arco	41.3	-	26.5	51.2	2	1.5	385
Rondônia							
Porto Velho	37.9	37.9-62.1	27.8	44.6	1	1.5	547
Ji-Paraná	62.1	-	72.2	55.4	2	2.2	584
Tocantins							
Tocantínia	30.9	30.9-70.3	9.7	45.1	1	2.8	527
Cristalândia	50.7	-	69.1	38.3	1	2.1	509

RR: relative risk.

Source: prepared by the authors, with data from: Brazilian Health Informatics Department ^3^; *SPI Amazon 2021* (Amazon Social Progress Index, https://painel.ipsamazonia.org.br/uploads/IPS_Amazonia_2023_dc7f4721ef.xlsx); Atmospheric Composition Analysis Group ^14^ and van Donkelaar et al. ^15^; Brazilian National Institute for Space Research ^8^.

Note: mortality rate per 100,000 inhabitants.

In Acre State, the index ranged from 40 to 77, with Santa Rosa do Purus showing the lowest index. In Amazonas State, the index varied from 39 to 82, with Fonte Boa presenting the lowest index. Moving to Rondônia State, Santa Luzia D’Oeste exhibited the lowest index at 38, whereas the state overall index ranged from 38 to 77. In Mato Grosso State, the index ranged from 45 to 86, with Comodoro showing the lowest index. Notably, except for Acre, the municipalities with the lowest index in Rondônia, Mato Grosso, and Amazonas states also scored lowest in the health and environment dimension (Table 3).

Shifting focus to Pará State, the municipality of Bannach showed the lowest index of 39, and the state overall index ranged from 39 to 68. In Tocantins, the index varied from 36 to 74, with Tocantínia presenting the lowest index. In Maranhão State, São Félix de Balsas exhibited the lowest index at 41, whereas the state overall index ranged from 41 to 73. In both Pará and Tocantins states, the municipalities with the lowest index scored poorly in the Education, Housing, and Sanitation dimension.

In Roraima State, São Luiz held the lowest index, ranging from 48 to 65 for the state. In Amapá State, the scanning analysis identified five municipalities, with Santana being designated as a priority due to its lowest index of 48. Similarly, in these states, municipalities with low indices also scored poorly in the Health and Environment dimension.

Table 4 and Figure 2b display the priority municipalities resulting from the cluster analysis of deaths in older adults caused by respiratory diseases. In the states of Acre, Amapá, and Roraima, only one municipality in each state − Rio Branco, Santana, and Uiramutã, respectively − was identified as an area with a high occurrence of deaths, making them priority municipalities in these states. We highlight that the indices were estimated separately for each state and for each outcome. Therefore, these mentioned priority municipalities, although part of significant clusters, do not present estimated indices, as there are no other municipalities for comparison in their respective states.

In Amazonas State, Japurá (34.9) showed the lowest index among the detected municipalities, with the Health and Environment dimension (27.1) playing a crucial role in defining the low index. Similarly, in Maranhão State, Peritoró (42.4) presented the lowest index, while in Mato Grosso State, Santo Antônio do Leverger (45.7) exhibited the lowest index. In Rondônia State, Porto Velho (37.9) held the lowest index, and in Pará State, Bannach (40.6) once again scored the lowest. In Tocantins, Tocantínia (30.9) remained the municipality with the lowest index. In Maranhão, Mato Grosso, Rondônia, Pará, and Tocantins states, the Education, Housing, and Sanitation dimension held significant importance in defining the municipalities with the lowest index in each state.

## Discussion

This study aimed to identify clusters with a high occurrence of hospitalizations due to respiratory diseases in children and adolescents, as well as deaths from respitaroy diseases in older adults in the Brazilian Legal Amazon, emphasizing priority municipalities based on estimated municipal indices for each state and analyzed outcome.

The analysis revealed clusters with varied temporal patterns for both hospitalizations and deaths across different periods. Generally, most hospitalization clusters occurred from 2009 to 2015, whereas mortality clusters were predominantly observed from 2014 to 2019. This finding corroborates data from the Brazilian Ministry of Health indicating a decrease in hospitalizations among children and adolescents alongside an increase in older adults mortality ^2^ ^,^ ^3^.

The demographic transition may have influenced the configuration of these clusters. The population aged over 60 in the Brazilian Legal Amazon increased from 7.3% in 2010 to 9.2% in 2019 ^17^. Moreover, approximately one-third of the Brazilian Legal Amazon total population resides in capitals and metropolitan areas ^18^. These factors likely contributed to a higher prevalence of first order mortality clusters towards the latter part of the period under analysis, and their locations in capitals/metropolitan regions.

The increased presence of hospitalization clusters early in the series could be attributed not only to demographic shifts but also to extreme climate events, such as droughts from 2009 to 2015, which may have contributed to the increased prevalence of resdpiratory diseases in certain areas. Moreover, the expansion of healthcare services over the period analyzed likely played a role in reducing hospitalizations. Rocha et al. ^17^ have outlined health improvements in the region, including the expansion of the Family Health Strategy (FHS), the fortification of riverine basic health units, and an increase in medical professionals numbers via the More Doctors Program ^17^. Carneiro et al. ^19^ further emphasized the FHS’s impact, suggesting a 28% drop in hospitalizations for ambulatory care-sensitive conditions among children in Pará from 2008 to 2017. We highlight that, despite these advancements, access to healthcare services continues to be a significant challenge in the region, especially for those residing outside metropolitan areas and with poor socioeconomic conditions.

The scan analysis identified two distinct patterns of clusters. The first occurred near or within capitals and metropolitan regions, where contributing factors included urban pollution, disorganized urbanization, poor socioeconomic conditions, and overwhelmed healthcare systems due to high population density. The second pattern was identified in areas farther from major centers, where health challenges included difficult access to adequate services due to long distances to healthcare facilities, inadequate infrastructure, a shortage of health professionals, and poor socioeconomic conditions ^17^.

The scanning analysis identified regions with persistent respiratory diseases-related issues, as highlighted in previous studies despite methodological differences. Rosa et al. ^20^ noted high hospitalization rates in the Tangará da Serra microregion (Mato Grosso State), a finding consistent with our analysis, which also marked this area as having a high occurrence of hospitalizations. Similarly, Rodrigues et al. ^21^, in their study across Rondônia State from 2001 to 2010, also identified municipalities in the central and southeastern parts of the state as high-incidence areas for respiratory diseases. Additionally, research by Andrade Filho et al. ^22^ in Rondônia State covering data from 2001 to 2012 highlighted Ji-Paraná municipality as a significant area for respiratory diseases mortality among older adults, corroborating our findings, which detected Ji-Paraná as a high mortality area from 2009 to 2012.

Some of the identified clusters are linked to intense deforestation and burning activities or are located near such areas ^8^. Studies conducted in the region reinforce the connection between wildfires and hospitalizations due to respiratory diseases, especially among children and older adults. Ignotti et al. ^23^, who assessed the impact of wildfires on hospitalization rates in the Amazon region from 2004 to 2005, observed an 8% and 10% increase in hospitalizations among children and older adults, respectively, during periods of higher PM_2.5_ concentration. According to Sant’Anna ^24^, in a more recent study, a total of 1,012 (95% confidence interval, 95%CI: 506-1,517) hospitalizations were attributable to respiratory diseases caused by wildfires in the entire Amazon biome territory from June to October 2019.

We highlight that wildfires are a significant risk factor for hospitalizations and deaths due to respiratory diseases, not only in the Brazilian Legal Amazon but across Brazil. Increased wildfire activity correlates with a higher rate of hospitalizations nationwide, especially in the northern region. Requia et al. ^25^ analyzed the impact of PM_2.5_ from wildfires on hospitalizations from 2008 to 2018, finding that wildfire periods are linked to a 23% (95%CI: 12%-33%) rise in respiratory disease hospitalizations across Brazil, and a 38% (95%CI: 30%-47%) increase in the northern region.

Moreover, Reddington et al. ^26^ have highlighted the broader health benefits of reducing wildfires in the Amazon. Their study, which assessed the impact of decreased wildfires from 2001 to 2012, a period marked by intensified government efforts to curb deforestation and wildfires, suggests that this reduction could have prevented from 400 to 1,700 premature deaths annually across South America. This underscores the widespread impact of wildfire smoke, which affect people’s health all over Brazilian Legal Amazon and beyond.

The estimation of composite synthetic indices enabled the observation of crucial factors related to population health beyond the estimated rates and relative risk. The Education, Housing, and Sanitation dimension played a significant role in prioritizing many municipalities within the detected clusters. Various factors influenced the health of the local population, extending the impact of smoke from wildfires even further. Climate factors, individual physiological characteristics such as age and pre-existing morbidities, income, access to education, healthcare services, and adequate housing all played vital roles in shaping population health and the formation of clusters. Thus, the decision to develop an index with more comprehensive indicators is important ^27^. In this context, it is essential to understand the interpretative nature of our synthetic indices. They function as aggregated indicators that reveal the strengths and weaknesses of municipalities in relation to the indicators used. This approach broadens our understanding of the municipalities, highlighting areas that require attention and providing a basis for future investigations and potential interventions.

A key characteristic of the scan model used in this study is the need to define a limit for the scanning window expansion. We chose the default software 50% limit for the at-risk population, a threshold that balances capturing a broad range of potential clusters while avoiding overextension into less relevant areas. To validate the robustness of our results, we also assessed different at-risk population percentages: 35%, 25%, and 10%. The consistency of these results with the default 50% confirms the stability of our findings. Additionally, a possible limitation is the choice of the scanning window shape. These windows can assume different shapes, such as circular and elliptical. The literature has already identified that the shape of the scanning windows can make a significant difference in detecting truly elongated clusters, especially for analyzing smaller and more sinuous regions ^28^. However, given that we are working with extensive state-level areas, and one of the objectives of these analyses is to identify important risk regions, we believe that a circular window would yield satisfactory results. Additionally, our analysis goes beyond the traditional scan statistics approach by incorporating the estimated municipality indices into the evaluation. This inclusion allows for a more comprehensive assessment.

## Conclusion

The scan analysis identified clusters across various time periods but they mostly aligned with the disease trends in the region. We found clusters both near metropolitan areas and in remote locations, capturing two distinct patterns of cluster distribution.

We highlight that this work of detecting clusters and prioritizing municipalities is an important starting point for directing research efforts to more specific regions, enabling the development of hypotheses and more complex studies that quantify the potential impacts of the aforementioned risk factors on their formation. Moreover, by identifying these clusters, we can better target resources and interventions to areas with the highest burden of respiratory diseases in children, adolescents, and older adults, thus contributing to more effective public health strategies in the Brazilian Legal Amazon region.
